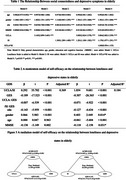# The Effects of subjective and objective lonelinesson Depression in elderly: The Mediating and Moderating Role of Self‐Efficacy

**DOI:** 10.1002/alz70857_097137

**Published:** 2025-12-24

**Authors:** Xiaoyan Pan, Caishui Yang

**Affiliations:** ^1^ Beijing Normal University, Beijing, Beijing, China

## Abstract

**Background:**

Old age is marked by a high prevalence of depressive symptoms, which impair cognitive performance. Loneliness and social isolation are modifiable risk factors for worsening depression, yet studies often focus on single constructs of loneliness. Research combining subjective and objective loneliness to explore their relationship with depression and the role of psycho‐motivation remains scarce. Integrated interventions addressing both loneliness and depression are essential.

**Method:**

A sample of 4295 Chinese elderly from the Beijing Aging Brain Renaissance Program (BABRI) was used to predict the risk of subjective and objective loneliness on the development of depressive symptoms in elderly by stratified binary logistic regression with the help of the UCLA Loneliness Scale and the Social Isolation Index, analyze the role of different self‐efficacies of elderly on subjective and objective loneliness on the occurrence of depressive symptoms by the mediation moderation analysis with the help of the Generalized Self‐Efficacy Scales (GSES).

**Result:**

After controlling for general characteristics, subjective and objective loneliness independently predicted depressive symptoms in the elderly (*p* <0.001), with higher social isolation increasing the risk of depression from subjective loneliness. Moderated models indicated that the product of self‐efficacy and subjective and objective loneliness were both significantly negatively correlated in elderly (B = ‐0.007, *p* =  0.000; B = ‐0.071, *p* =  0.000). Self‐efficacy had a positive effect on subjective and objective loneliness in elderly and further influenced their depressive symptoms. In addition, self‐efficacy partially mediated the association between subjective and objective loneliness and depressive symptoms, with indirect effects of 0.481 and 0.143, respectively.

**Conclusion:**

From different dimensions of loneliness, this study explored the predictive roles of subjective loneliness (emotional) and objective loneliness (behavioral) in the risk of depressive symptoms in the elderly. It provides a reference basis for preventing depressive symptoms in the elderly from developing into clinical disorders from the perspective of loneliness. Additionally, it emphasizes that self‐efficacy is an important mediating and moderating mechanism in the impact of subjective loneliness on depression, highlighting the necessity of targeted interventions for loneliness in different elderly populations.